# Analysis of copy number variations and candidate genes in recurrent pregnancy loss

**DOI:** 10.7717/peerj.20889

**Published:** 2026-03-03

**Authors:** Luming Wang, Li Yang, Suping Li, Xiaodan Liu, Ping Tang

**Affiliations:** Fetal Medicine Center, Jiaxing Maternity and Child Health Care Hospital, Jiaxing, Zhejiang, China

**Keywords:** Recurrent pregnancy loss, Copy number variation, Whole exome sequencing, CNV-seq, SNP-array

## Abstract

**Background:**

Recurrent pregnancy loss (RPL) is often associated with genetic factors. This study investigates chromosomal abnormalities in RPL by analyzing copy number variations (CNVs) and single-nucleotide variants (SNVs).

**Methods:**

We conducted a retrospective analysis of 400 RPL patients, with 393 successfully analyzed using CNV-seq and single nucleotide polymorphism (SNP)-array after excluding maternal cell contamination (MCC). Additionally, 16 families with normal results underwent whole exome sequencing (WES).

**Results:**

Among the patients, 187 (47.6%) showed normal results, while 206 (52.4%) exhibited abnormalities, including 152 aneuploidies (73.8%), 37 CNVs (18.0%), and 17 triploidies (8.3%). Statistical analysis revealed a significant increase in chromosomal abnormalities with advancing maternal age, but no significant differences in rates were observed before 24 weeks of pregnancy in patients with two or more miscarriages. We identified 28 pathogenic (P)/likely pathogenic (LP) CNVs and six P/LP SNVs, implicating 808 morbid genes. Enrichment analysis and protein-protein interaction (PPI) network construction revealed 69 key genes in critical pathways, with *IL6*, *TNF*, and *ACTB* as hub genes.

**Discussion:**

These findings contribute to establishing genetic markers for RPL screening in the Chinese population, enhancing our understanding of miscarriage etiology and facilitating prenatal diagnosis.

## Introduction

The clinical incidence of spontaneous abortion is about 15%, with recurrent pregnancy loss (RPL) occurring in around 5%, a figure that has shown an upward trend in recent years (Dimitriadis et al., 2020). Internationally, the definition of RPL varies in regard to the number of occurrences, gestational week limit, consecutive events, and the inclusion of biochemical pregnancies.

The Royal College of Obstetricians and Gynaecologists (RCOG) defines RPL as the consecutive loss of three or more pregnancies before 24 weeks, including biochemical pregnancies (Regan et al., 2023). The American Society for Reproductive Medicine (ASRM) standardizes RPL as two or more spontaneous abortions, requiring ultrasound or histological confirmation, excluding biochemical pregnancies (Practice Committee of the American Society for Reproductive Medicine, 2012). The European Society of Human Reproduction and Embryology (ESHRE) characterizes RPL as two or more pregnancy failures before 24 weeks, confirmed by blood or urine hCG, and this includes biochemical pregnancies (Bender Atik et al., 2018).

Studies indicate no significant difference in detecting causative factors between those with a history of two or three or more recurrent miscarriages (Goldstein et al., 2017). Therefore, in this study, RPL is defined as the consecutive occurrence of two or more pregnancy losses before 24 weeks with the same spouse, including biochemical pregnancies.

Recurrent miscarriage imposes significant physiological and psychological stress on patients and presents substantial challenges for doctors. The etiology of RPL is highly intricate, and the distribution of contributing factors varies with the number and timing of previous miscarriages. Major factors include chromosomal or genetic abnormalities, disruptions in the endocrine system, immune dysfunction, uterine structural anomalies, thrombophilic conditions, infections, and environmental factors (Alecsandru et al., 2021). Existing research indicate that approximately 50% of RPL cases can be attributed to genetic factors, such as aneuploidy or triploidy (Stephenson, Awartani & Robinson, 2002). Genetic testing and analysis of products of conception hold crucial value in determining the causes of miscarriage, assessing recurrence risk, and facilitating prenatal diagnostics.

Among genetic causes, balanced chromosomal abnormalities represent one of the major contributors to RPL (Li et al., 2022). These structural rearrangements, most commonly reciprocal or Robertsonian translocations and inversions, do not involve significant gain or loss of genetic material, and carriers are usually phenotypically normal. However, during meiosis, abnormal pairing and segregation of the rearranged chromosomes can lead to the formation of unbalanced gametes, which may result in early embryonic loss. Retrospective studies have shown that most chromosomal abnormalities detected in RPL couples are balanced rearrangements, predominantly reciprocal translocations, and corresponding chromosomal deletions and duplications can often be identified in the products of conception using single nucleotide polymorphism (SNP)-array analysis (Aynaci et al., 2025). In addition, recent studies have revealed cryptic balanced translocations that are undetectable by conventional karyotyping but can be identified using fluorescence *in situ* hybridization (FISH) (Bahap et al., 2025). These findings emphasize the importance of combining cytogenetic and molecular methods, such as FISH and SNP-array, to improve the detection efficiency of structural chromosomal abnormalities in RPL.

In recent years, the investigation of RPL has advanced significantly with the application of high-resolution molecular techniques, including chromosomal microarray analysis (CMA) and next-generation sequencing (NGS). Beyond conventional chromosomal numerical abnormalities, certain copy number variations (CNVs) have been recognized as potential contributors to miscarriage (Bai et al., 2023). Additionally, the use of whole exome sequencing (WES) for detecting single nucleotide variants (SNVs) in high-risk pedigrees has revealed associations between severe monogenic disorders and RPL (Liu et al., 2024). While these studies illuminate specific causes of RPL, the overall sample size is relatively small, limiting the conclusive identification of candidate genes and related biological processes. Further extensive research is necessary to fill these gaps in our understanding.

To systematically investigate chromosomal abnormalities in RPL, we analyzed 400 patients using single nucleotide polymorphism array (SNP-array) and copy number variation sequencing (CNV-seq). For the 16 patients without detected abnormalities, trio-WES was employed to identify candidate genes associated with RPL. Combining OMIM-morbid genes identified in CNVs and SNVs, we further analyzed gene functions, utilizing gene enrichment and protein interaction, to understand critical genes in embryonic development. These findings may contribute to establishing a genetic marker-based screening for RPL in the Chinese population, offering valuable guidance for high-risk individuals.

## Materials & Methods

### Study subjects

A prospective study was conducted at the Fetal Medicine Center of Jiaxing Maternity and Child Health Care Hospital from 2019 to 2024. Inclusion criteria comprised: (1) miscarriage occurring before 24 weeks of gestation; (2) two or more consecutive miscarriages with the same spouse, including biochemical pregnancies; (3) absence of apparent abnormalities in pregnant women. Exclusion criteria included: (1) pregnant women with genital abnormalities or significant diseases such as immune, endocrine, or infectious conditions; (2) subjective requests for pregnancy termination; (3) inadequate quality of miscarriage specimens. Both spouses were informed about the advantages and limitations of the testing, agreed to participate, and signed written informed consent. This study was approved by the Ethics Committee of Jiaxing Maternity and Child Health Care Hospital, approval number 2019 (Ethics)-48.

To ensure adequate statistical power, the sample size was estimated using the formula n = z^2^p(1−p)/d^2^, where *z* = 1.96 (95% confidence level), *p* = 0.5 (expected proportion), and *d* = 0.05 (margin of error). The minimum required sample size was calculated as 385 cases. Considering potential exclusions such as maternal cell contamination or inadequate DNA quality, a total of 400 samples were included, of which 393 yielded analyzable results, satisfying the statistical requirement for robustness and reproducibility.

**Figure 1:**
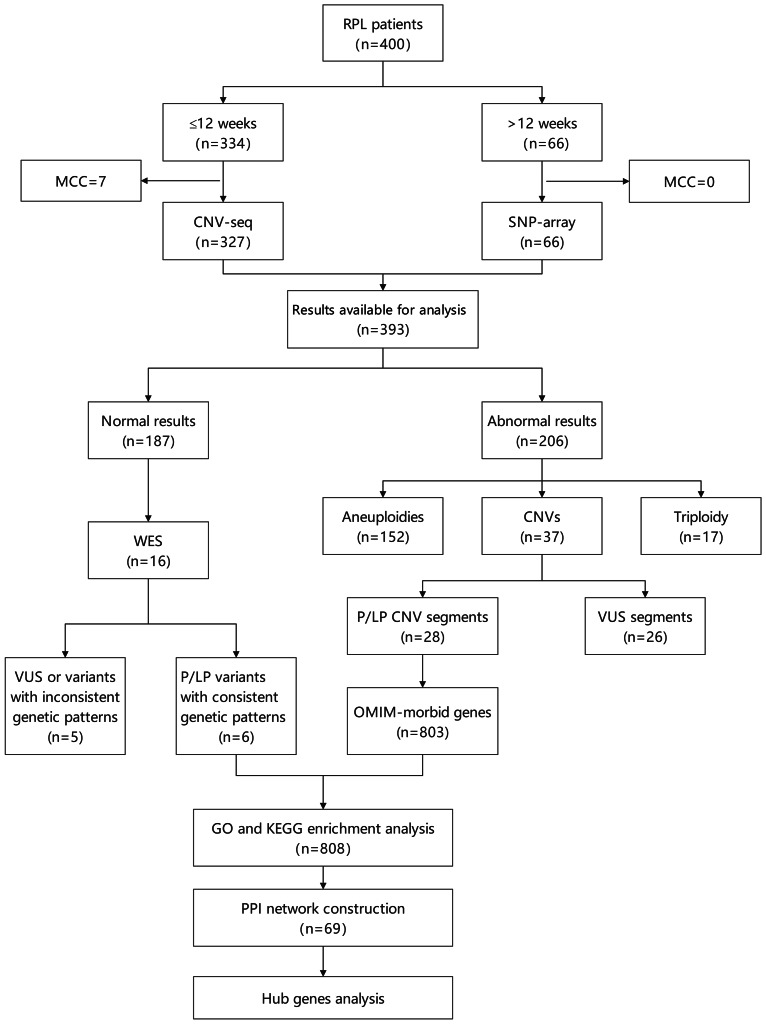
Flow diagram illustrating the miscarriage patients included in the study and the analytical strategies employed. RPL, recurrent pregnancy loss; MCC, maternal cell contamination; SNP-array, single nucleotide polymorphism array; CNV-seq, copy number variation sequencing; WES, whole exome sequencing; P/LP, pathogenic/likely pathogenic; VUS, variant of uncertain significance; PPI, protein–protein interaction.

The inclusion samples, consisting of chorionic villus or fetal tissue, were rinsed with physiological saline and then subjected to DNA extraction using the QIAamp DNA Mini Kit (Qiagen, Hilden, Germany). Peripheral blood samples from both parents, collected with EDTA anticoagulant, were also extracted using the QIAamp DNA Blood Mini Kit (Qiagen, Hilden, Germany) for maternal cell contamination (MCC) identification and trio-WES. MCC was identified using the short tandem repeat (STR) method with the Human STRtyper-21G fluorescent detection kit (HEALTH Gene Technologies, Ningbo, China), analyzed on the ABI 3500Dx Genetic Analyzer (Applied Biosystems, Foster City, CA, USA). Subsequent experimental procedures followed after excluding MCC, as detailed in Fig. 1.

### CNV-seq

The genomic DNA was fragmented and subjected to end-repair facilitated by restriction enzymes. Adapter sequences, bearing labeling tags, were ligated to the ends of DNA strands using ligases. Following PCR amplification and purification, the processed DNA fragments were assembled into a library. The library then underwent a series of procedural steps, including single-strand separation, circularization, and rolling circle replication, to generate DNA nanoballs. Subsequently, sequencing was conducted using the combinatorial probe-anchor synthesis (cPAS) method on the MGISEQ-2000 sequencer (BGI, Shenzhen, China). Raw data underwent preprocessing steps, such as sequence alignment, deduplication, and GC correction. CNV analysis was then performed using statistical algorithms to derive the results.

### SNP-array

This study employed the CytoScan 750K Suite (Thermo Fisher Scientific, Waltham, MA, USA) CMA platform for comprehensive analysis. The CytoScan 750K array, with approximately 550,000 oligonucleotide probes and 200,000 SNP probes spaced at an average of 4.1 kb, facilitated a whole genome scan. Following the manufacturer’s protocol, genomic DNA was fragmented, labeled, and hybridized onto the chip array, followed by washing and scanning to obtain raw data. Chromosome analysis, detection of CNVs, and identification of regions of allelic homozygosity (ROHs) were performed using ChAS 4.3 software (Thermo Fisher Scientific).

### Evaluation of CNVs

Variants were annotated and interpreted using multiple databases (DGV, DECIPHER, ClinGen, OMIM, PubMed, *etc.*). According to the guidelines of the American College of Medical Genetics and Genomics (ACMG) and the Clinical Genome Resource (ClinGen), variants were classified into five categories: (1) pathogenic (P) CNVs; (2) likely pathogenic (LP) CNVs; (3) variants of uncertain significance (VUS); (4) likely benign (LB) CNVs; and (5) benign (B) CNVs. This study exclusively reports P/LP CNVs and VUS, while excluding B/LB CNVs.

### WES

Genomic DNA underwent fragmentation, magnetic beads selection, end repair, adapter ligation, PCR amplification, and purification to construct the library. Customized Roche KAPA HyperExome capture probes were employed for efficient enrichment of human exome regions. The hybridized libraries underwent single-strand separation, circularization, and rolling circle replication to generate DNA nanoballs, which were sequenced using the MGISEQ-2000 sequencer (BGI, Shenzhen, China).

The raw data were processed with SOAPnuke to obtain clean reads, which were then aligned to the hg19 reference genome using BWA software. SNV and Indel detection were conducted with GATK software, while CNV analysis utilized exomeDepth and CNVkit. Following annotation in multiple databases, variants were interpreted for pathogenicity according to guidelines of ACMG.

### Statistical analysis

Statistical data were described as mean ± SD. Differences between groups were analyzed using the chi-square test or Fisher’s exact test. A *p*-value <0.05 was considered statistically significant. Data analysis was conducted using SPSS software, version 19.0 (IBM Corp., Armonk, NY, USA).

### Functional enrichment analysis

We obtained a list of OMIM-morbid genes within the P/LP CNV segments from the UCSC Genome Browser. Enrichment analysis was then conducted using Gene Ontology (GO) and Kyoto Encyclopedia of Genes and Genomes (KEGG), incorporating the P/LP genes detected by WES. Statistical significance for enrichment was defined as an adjusted *p*-value <0.05. Candidate genes from significant GO and KEGG results were selected to construct a protein-protein interaction (PPI) network using the String database within Cytoscape software. Further prioritization of candidate genes was performed using the cytoHubba plugin to identify hub genes closely associated with embryonic development.

## Results

### Patients characteristics and analysis of results

**Figure 2:**
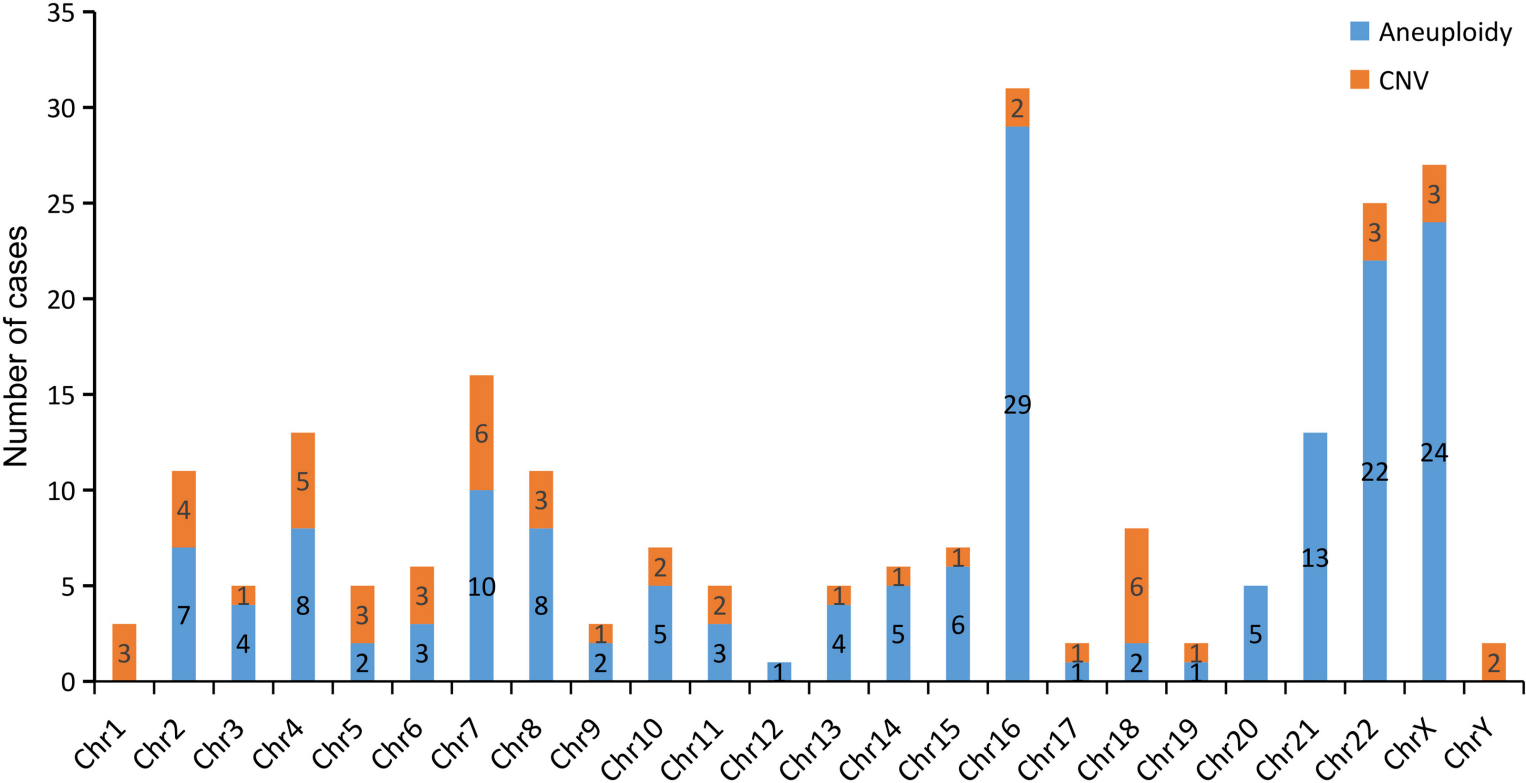
Distribution of aneuploidy and CNV abnormalities across different chromosomes.

Among the 400 patients, the mean maternal age was 29.73 ±  4.57 years. The mean gestational weeks at miscarriage was 9.87 ± 2.74, with an average of 2.55 ±  0.89 miscarriages per woman. In these patients, 334 miscarriages (83.5%) occurred at ≤12 weeks; after excluding 7 patients with MCC, 327 patients were successfully tested using CNV-seq. For the 66 patients (16.5%) >12 weeks, all were tested using SNP-array with no MCC. Overall, 393 results were analyzed, with 187 (47.6%) normal and 206 (52.4%) abnormal. Among the normal results, 16 patients underwent further WES analysis. Abnormal results included 152 aneuploidies (73.8%), 37 CNVs (18.0%), and 17 triploidies (8.3%). Figure 2 shows the chromosomal distribution, with most abnormalities on chromosomes 16, 22, and X, with predominant findings of trisomy 16, trisomy 22, and monosomy X.

Statistical analysis was conducted on the frequency of chromosomal abnormalities among different groups based on age, gestational weeks, and number of miscarriage (Table 1). The abnormality rate was around 50% for women under 30 and those aged 30–34. For women aged 35–39 and those aged ≥40, the abnormality rate increased to around 70%. The rate of chromosomal abnormalities significantly increased with age (*P* = 0.032). Before 24 weeks of gestation, the difference in the rate of chromosomal abnormalities among different gestational weeks was not statistically significant. Similarly, the rate of chromosomal abnormalities increased with the number of miscarriages, but the difference was not statistically significant.

**Table 1:** Frequency of chromosomal abnormalities across different groups categorized by age, gestational weeks, and number of miscarriages.

Characteristics	Classification	Total cases (n)	Frequency (%)	Abnormal cases (n)	Frequency (%)
Age	<30	211	53.7	103	48.8
	30–34	121	30.8	61	50.4
	35–39	44	11.2	30	68.2
	≥40	17	4.3	12	70.6
Gestational weeks	<8	55	14.0	27	49.1
	8–12	272	69.2	149	54.8
	13–17	60	15.3	28	46.7
	≥18	6	1.5	2	33.3
Number of miscarriages	2	250	63.6	125	50.0
	3	95	24.2	51	53.7
	4	30	7.6	18	60.0
	≥5	18	4.6	12	66.7

### Results of CNVs evaluation

Among the 37 patients with detected CNVs, a total of 54 segments were identified, including 28 P/LP CNV segments and 26 VUS. The P/LP CNVs were distributed across various chromosomes (Table 2). The average age of the 20 patients with P/LP CNVs was 29.95 ± 4.48 years, with a mean gestational weeks of 9.85 ± 2.01 and an average of 2.70 ± 0.87 miscarriages. Of these CNVs, 18 were large CNVs (>10 Mb) and 10 were submicroscopic CNVs (<10 Mb). Patients 004, 005, 009, 010, and 014 are presumed to have parents carrying reciprocal translocations, among whom patients 004, 005, and 009 were confirmed to have maternal balanced translocations with karyotypes 46,XX,t(13;14)(q13;q24), 46,XX,t(5;7)(p14;p12), and 46,XX,t(16;19)(q13;q13.4), respectively. Patients 012 and 015 are presumed to have parents carrying inversions, with patient 015 confirmed to have a paternal inversion, karyotype 46,XY,inv(6)(p21.3q25.3).

**Table 2:** Clinical characteristics of fetuses with detected pathogenic and likely pathogenic CNVs.

Patient ID	Age	Gestational weeks	Number of miscarriages	Cytoband	Type	Genomic coordinates	Size (Mb)	Related disease
001	29	14	3	2q34q37.3	Dup	Chr2: 209,321,854–240,443,548	31.12	Partial trisomy 2q
				7q31.1q36.3	Dup	Chr7: 110,459,063–159,119,707	48.66	Partial trisomy 7q
002	25	14	3	Xp22.31p22.31	Del	ChrX: 6,427,993–8,147,809	1.72	X-linked ichthyosis
003	27	13	2	22q11.21q11.21	Del	Chr22: 18,914,279–21,574,970	2.66	DiGeorge syndrome
004	28	8	3	13q14.2q34	Del	Chr13: 48,309,942–115,109,828	66.80	13q deletion syndrome
				14q31.1q32.33	Dup	Chr14: 79,583,616–107,259,901	27.68	Partial trisomy 14q
005	29	10	3	5p14.3p15.33	Del	Chr5: 10,429–21,482,568	21.47	Cri-du-chat syndrome
				7p12.1p22.3	Dup	Chr7: 53,467–52,076,677	52.02	Trisomy 7p
006	27	8	2	18p11.31p11.32	Del	Chr18: 138,005–6,596,177	6.46	18p terminal deletion
007	30	10	4	4p16.1p16.3	Del	Chr4: 10,004–9,583,036	9.57	Wolf-Hirschhorn syndrome
008	29	11	2	16p13.11	Del	Chr16: 15,050,139–16,469,743	1.42	16p13.11 recurrent microdeletion
009	24	8	2	19q13.42q13.43	Del	Chr19: 55,663,201–59,060,529	3.40	19q13 microdeletion syndrome
				16q12.2q24.3	Dup	Chr16: 56,068,070–90,261,674	34.19	Partial trisomy 16q
010	32	7	2	8p11.1p23.3	Del	Chr8: 10,132–43,351,205	43.34	Deletion 8p syndrome
				11q24.2q25	Dup	Chr11: 124,500,019–134,946,238	10.45	Partial trisomy 11q
011	41	10	2	17p11.2p12	Del	Chr17: 15,282,392–18,885,526	3.60	Smith-Magenis syndrome
012	37	9	2	8p23.3p11.23	Del	Chr8: 10,132–37,726,519	37.72	8p23.1 deletion syndrome
				8p11.21q24.3	Dup	Chr8: 40,004,124–146,303,892	106.30	Trisomy 8q
013	28	9	3	5p15.33p13.3	Del	Chr5: 1–29,580,810	29.58	Cri-du-chat syndrome
014	31	8	2	3q26.33q29	Dup	Chr3: 179,206,479–197,946,596	18.74	3q29 microduplication syndrome
				18p11.32p11.31	Del	Chr18: 138,005–3,805,674	3.67	18p terminal deletion
015	33	9	3	6q27	Del	Chr6: 165,048,606–171,050,010	6.00	6q27 terminal deletion
				6p25.3p21.33	Dup	Chr6: 63,810–31,655,857	31.59	Partial trisomy 6p
016	29	9	2	6p25.3p21.32	Dup	Chr6: 1–32,596,197	32.60	Partial trisomy 6p
017	37	11	2	7p21.1p15.1	Dup	Chr7: 17,511,510–28,494,032	10.98	Partial trisomy 7p
018	23	11	4	9q21.31q31.1	Del	Chr9: 83,725,203–106,398,261	22.67	Au-Kline syndrome
019	32	10	3	22q11.21q11.21	Dup	Chr22: 18,765,311–21,746,118	2.98	22q11 duplication syndrome
020	28	8	5	1q31.2q44	Dup	Chr1: 193,563,341–249,224,684	55.66	Partial trisomy 1q

**Notes.**

Dup duplication Del deletion

### Analysis of WES results

Trio-WES was performed on 16 families, identifying 11 candidate genes, with six P/LP variants showing consistent genetic patterns (Table 3). Four novel variants were identified in this study:

**Table 3:** Clinical characteristics of fetuses with detected pathogenic and likely pathogenic SNVs.

Patient ID	Gene	Genomic coordinates	Variant	Genotype (fetus/father/ mother)	Pathogenicity	Related disease/inheritance	Supplementary phenotype	References
01	*GATA4*	chr8:11566254	NM_001308093.3:c.433G>T (p.G145C)	Het/WT/WT	LP	Congenital heart defects/AD	NA	This study
02	*COL5A2*	chr2:189963452	NM_000393.3:c.402+1G>A	Het/Het/WT	P	Ehlers-Danlos syndrome/AD	Fetal increased nuchal translucency (NT) with cervical lymphatic hygroma; father’s joint hypermobility and scoliosis	This study
03	*RAPSN*	chr11:47469615	NM_005055.4:c.280G>A (p.Glu94Lys)	Hom/Het/Het	LP	Fetal akinesia deformation sequence/AR	Fetal multiple anomalies and hydrops	Natera-de Benito et al. (2016); Natera-de Benito et al. (2017); Müller et al. (2007); Qi et al. (2020)
04	*PPIB*	chr15:64448943	NM_000942.4:c.509G>A (p.Gly170Asp)	Hom/Het/Het	LP	Osteogenesis imperfecta/AR	Fetal long bones of the limbs are shortened (<-2SD)	Zhu et al. (2021); Chang et al. (2020); Jiang et al. (2017)
05	*GREB1L*	chr18:19088166-19088167	NM_001142966.1:c.4457_4458delAA (p.Lys1486Argfs*42)	Het/Het/WT	P	Renal hypodysplasia/aplasia/AD	Absence of amniotic fluid; fetal kidneys not clearly visible	This study
06	*SIX3*	chr2:45169350	NM_005413.3 (46,XN,inv(2)(p21p21))	Het/Het/WT	LP	Holoprosencephaly/AD	Fetal brain development abnormality and possible holoprosencephaly	This study

**Notes.**

Het heterozygous Hom homozygous WT wild type P pathogenic LP likely pathogenic AD autosomal dominant AR autosomal recessive

1. *GATA4*, NM_001308093.3:c.433G>T (p.G145C): A *de novo* missense mutation. During fetal development, *GATA4* is expressed in the yolk sac endoderm and heart-forming cells (Arceci et al., 1993). Variants in *GATA4* can cause atrial septal defect, ventricular septal defect, and Tetralogy of Fallot. ACMG classification: LP, evidence: PM1+PM2+PP2+PP3.
2. *COL5A2*, NM_000393.3:c.402+1G>A: A splice site mutation inherited from the father. Variants in *COL5A2* lead to Ehlers-Danlos syndrome with variable phenotypes and severity. ACMG classification: P, evidence: PVS1+PM2+PP3.
3. *GREB1L*, NM_001142966.1:c.4457_4458delAA (p.Lys1486Argfs*42): A frameshift mutation inherited from the father. Variants in *GREB1L* cause renal hypodysplasia/aplasia with high phenotypic variability and incomplete penetrance (Sanna-Cherchi et al., 2017). Affected individuals may show severe renal developmental abnormalities *in utero*, which can be fatal (Brophy et al., 2017). ACMG classification: P, evidence: PVS1+PM2+PP3.
4. 46,XN,inv(2)(p21p21).seq[GRCh37/hg19](45169350-47173796): An inversion of approximately 2 Mb in the 2p21 region, with a breakpoint at chr2:45169350 in exon 1 of the *SIX3* gene. Variants in *SIX3* lead to holoprosencephaly with variable severity and incomplete penetrance (Mercier et al., 2011). ClinGen classification: LP, Haploinsufficiency (HI) Score: 3.

### Functional enrichment analysis results

**Figure 3:**
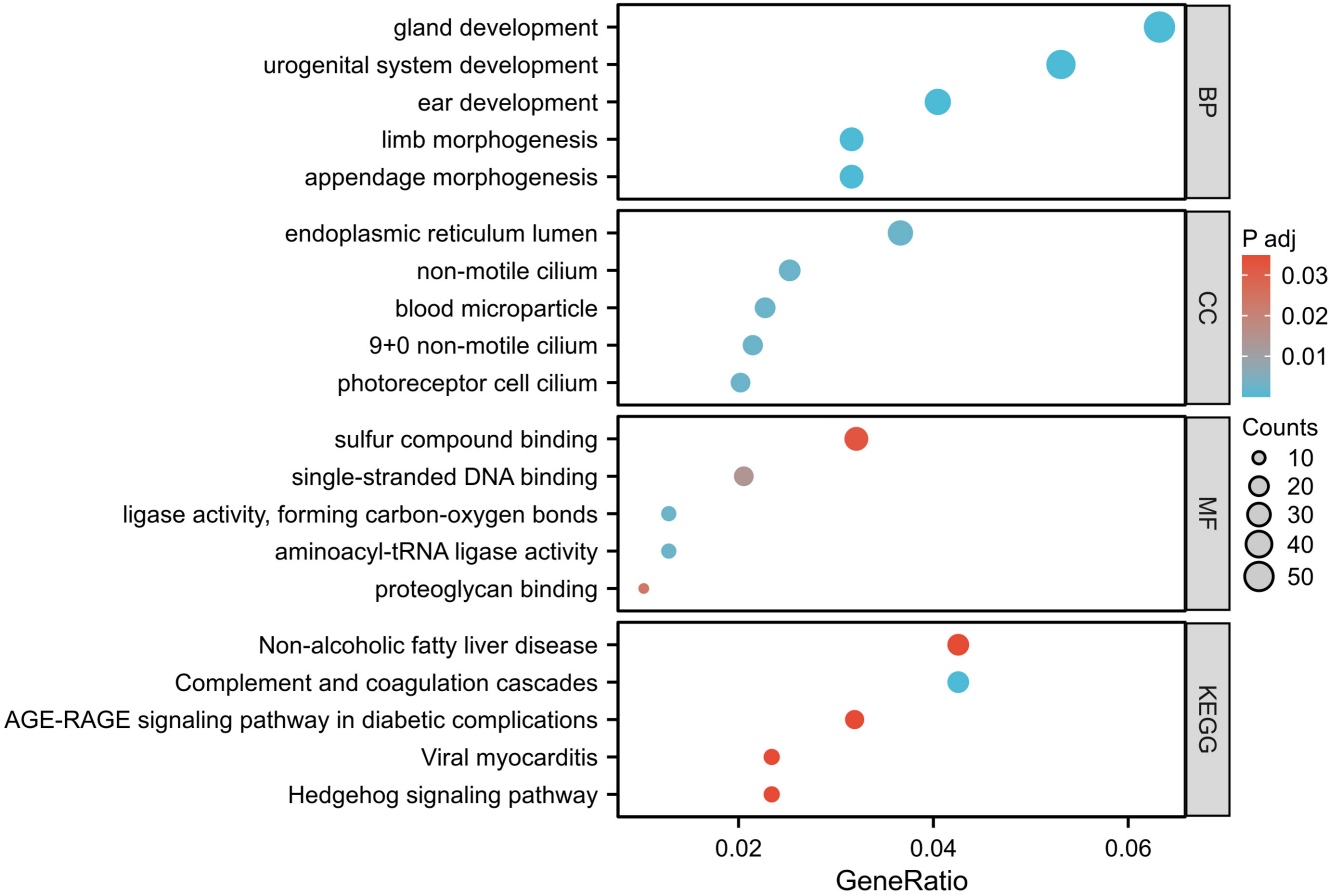
Top five enriched pathways identified through GO and KEGG enrichment analyses, with adjusted *p*-values < 0.05.

**Figure 4:**
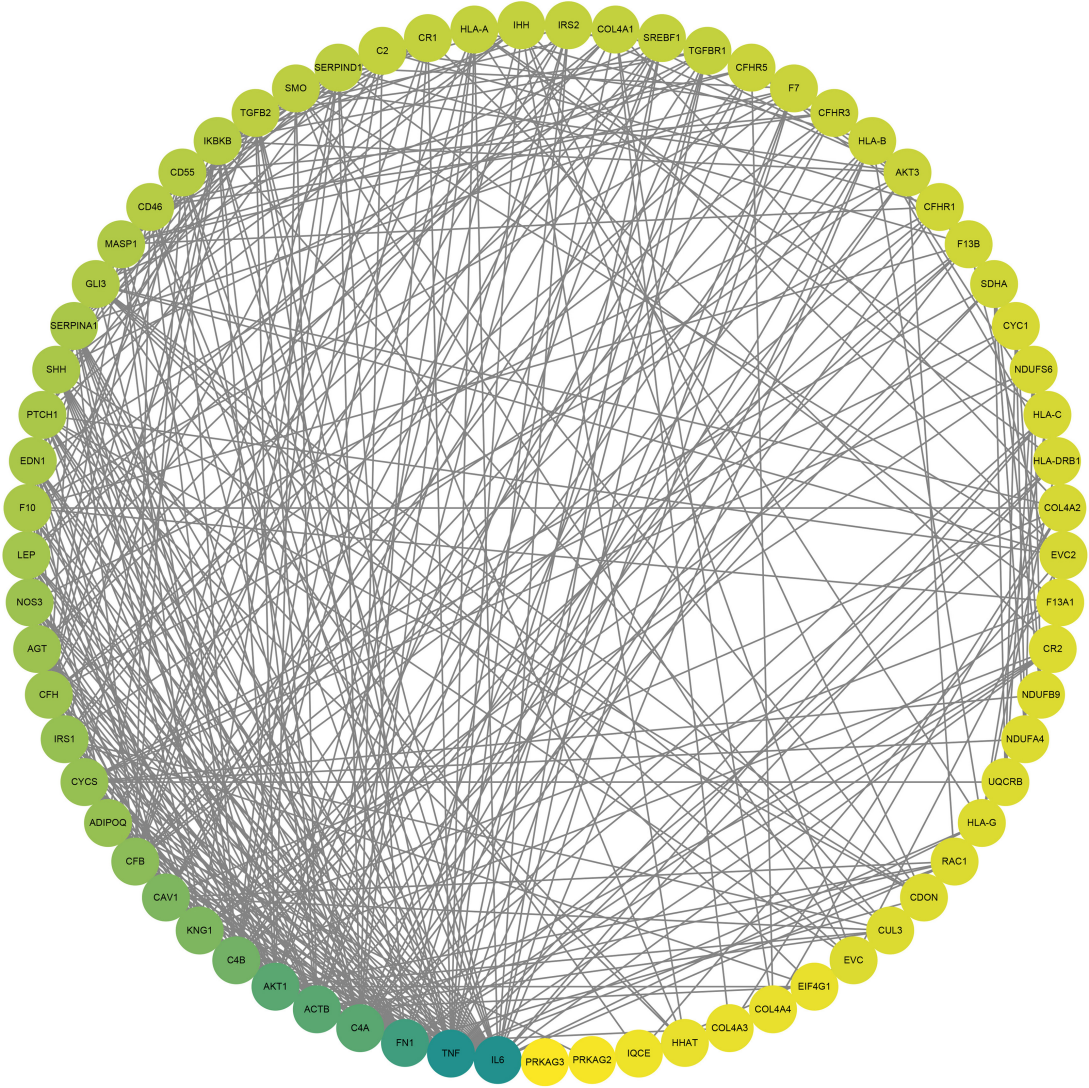
PPI network diagram of potential candidate genes in related signaling pathways. Node color intensity indicates the degree of connectivity, with darker green representing higher connectivity. The network includes 69 nodes and 410 edges, highlighting the top five genes: *IL6*, *TNF*, *FN1*, *C4A*, and *ACTB*.

By extracting OMIM-morbid genes from the P/LP CNV segments, 803 related genes were identified. Including P/LP genes detected by WES, a total of 808 candidate genes were listed. GO and KEGG enrichment analyses revealed statistically significant results (p.adjust <0.05), as shown in Fig. 3. The enriched pathways were primarily associated with embryonic and urogenital system development, cilium organization, and immune or inflammatory responses. Notably, KEGG analysis revealed significant enrichment in the Complement and coagulation cascades and Hedgehog signaling pathways, both of which have been implicated in early embryonic development and pregnancy maintenance. A PPI network was constructed for 69 key genes from these pathways, with the top five genes identified as *IL6*, *TNF*, *FN1*, *C4A*, and *ACTB* (Fig. 4). Using the CytoHubba plugin, the top 10 nodes were ranked by the Maximal Clique Centrality method, identifying the top five genes: *IL6*, *TNF*, *AKT1*, *ACTB*, and *CAV1*. The intersection of these two sets identified the hub genes *IL6*, *TNF*, and *ACTB*.

## Discussion

RPL exerts significant psychological and financial burdens on affected patients (Quenby et al., 2021). More than 80% of spontaneous miscarriages occur before 12 weeks of gestation, which aligns with the 83.5% observed in this study. Fetal chromosomal abnormalities, including large segment deletions and duplications, are key factors contributing to early pregnancy loss (Wang et al., 2020). CNV-seq, a NGS method based on low-depth whole-genome sequencing, enables variable resolution by adjusting sequencing depth (Zhang et al., 2021). In this study, 1M resolution CNV-seq was employed for detecting RPL patients before 12 weeks, offering advantages such as broad detection range, high throughput, ease of operation, good compatibility, and low DNA input requirements (Liang et al., 2014). For patients between 13 and 24 weeks of gestation, a 100 Kb resolution SNP-array was utilized. This tiered testing approach is highly cost-effective, enhancing efficiency without compromising diagnostic accuracy and reducing the financial burden on patients. For patients with unexplained RPL requiring further investigation, Trio-WES was applied to explore single gene variants. Studies show that WES identifies gene variants linked to RPL (Tsurusaki et al., 2014; Filges et al., 2015; Qiao et al., 2016), enhancing understanding of embryonic development and guiding preimplantation genetic diagnosis.

The incidence of chromosomal abnormalities increases with maternal age, likely due to functional changes associated with oocyte aging, which is consistent with previous studies (Chiang, Schultz & Lampson, 2012). No significant differences in chromosomal abnormality rates were observed before 18 weeks of gestation, after which the rates decreased (Table 1). Some studies have shown that as gestational age increases, the incidence of chromosomal abnormalities gradually decreases, reaching its lowest after 28 weeks (Lan et al., 2021). This is consistent with the above results. In this study, an increase in the number of miscarriages was associated with a higher rate of chromosomal abnormalities, but the difference was not statistically significant. Other studies have indicated that an increased number of miscarriages does not significantly alter the rate of chromosomal abnormalities, and may even decrease the likelihood of chromosomal abnormalities in embryos (Gomez et al., 2021). This suggests that the relationship between miscarriage frequency and chromosomal abnormalities may not be linear and is influenced by multiple factors (Zhu et al., 2023).

In this study, aneuploidies and CNVs were detected across all chromosomes, demonstrating extensive coverage. The high incidence of aneuploidies on chromosomes 16, 22, and X is consistent with findings from other studies (Sheng et al., 2021). Monosomy X leads to Turner syndrome, with an incidence of approximately one in 2,500 live-born females. Around 99% of fetuses with a 45, XO karyotype undergo spontaneous miscarriage during the first or second trimester (Gravholt et al., 2023), which aligns with the results of this study. Some of the P/LP CNVs identified in this study, such as those associated with X-linked ichthyosis, DiGeorge syndrome, Wolf-Hirschhorn syndrome, 16p13.11 recurrent microdeletion, Smith-Magenis syndrome, Cri-du-chat syndrome, Au-Kline syndrome, and 22q11 duplication syndrome, do not typically result in miscarriage or cessation of embryonic development. The reasons may include the presence of other unidentified factors contributing to miscarriage or the limited sample size, leading to heterogeneity in individual abnormal patients. Additionally, chromosomal structural abnormalities indicated by other CNVs are also important contributors to miscarriage (Elhady, Kholeif & Nazmy, 2020).

Among the six P/LP variants identified through WES, two have been previously reported. The NM_005055.4(*RAPSN*):c.280G>A (p.Glu94Lys) and NM_000942.4 (*PPIB*):c.509G>A (p.Gly170Asp) mutations were homozygous, inherited from carrier parents, and followed an autosomal recessive inheritance pattern. Studies have shown that partial loss of RAPSN function can lead to congenital myasthenia (Müller et al., 2007), while severe functional loss can result in a lethal fetal akinesia phenotype (Vogt et al., 2008). The most common *RAPSN* compound heterozygous mutations, c.264C>A (p.Asn88Lys) and c.280G>A (p.Glu94Lys), have been associated with decreased fetal movements and postnatal hypotonia, poor sucking, and neck muscle weakness, indicating congenital myasthenic syndrome (CMS) (Natera-de Benito et al., 2016; Natera-de Benito et al., 2017). Another patient reported a fetus with compound heterozygous *RAPSN* mutations (c.280G>A and c.288delG) presenting with subcutaneous edema and hydrothorax, leading to pregnancy termination (Qi et al., 2020). The homozygous *RAPSN* variant identified in this study, associated with severe intrauterine abnormalities, has not been previously reported, providing new clinical evidence for studying *RAPSN*’s pathogenic mechanisms. The *PPIB* mutation c.509G>A (p.Gly170Asp) is linked to osteogenesis imperfecta (OI) type IX, a condition that can be lethal in the perinatal period (Pyott et al., 2011). This mutation has been reported to cause skeletal abnormalities in fetuses, with a founder effect identified in the Chinese population (Zhu et al., 2021; Chang et al., 2020).

A total of 808 morbid genes were implicated in the identified P/LP CNVs and SNVs. Through a series of enrichment analyses and rankings, the potential hub genes *IL6*, *TNF*, and *ACTB* were identified. *IL6*, a type of interferon (IFN), is evolutionarily conserved and upregulated during embryo implantation (Griffith et al., 2017). In mouse models, it has been shown to induce autism-like phenotypes and abnormal brain development (Smith et al., 2007). *TNF*, a cytokine, is toxic to early embryonic development (Robertson et al., 2018) and has been found to induce preterm birth in non-human primate models (Sadowsky et al., 2006), while mediating fetal death in mice (Carpentier, Dingman & Palmer, 2011). Research has demonstrated that abnormal expression of cytokines and interferons is a common and critical mediator of congenital diseases and fetal loss in patients with infection, chromosomal abnormalities, metabolic, and autoimmune diseases (Yockey & Iwasaki, 2018). The *ACTB* gene encodes *β*-actin, and its mRNA regulates embryogenesis, differentiation, and cell migration (Cuvertino et al., 2017; Hüttelmaier et al., 2005). Studies have shown that *ACTB*, as a housekeeping gene, is stably expressed in the endometrial tissues of women with RPL (Stocker, Cagampang & Cheong, 2017).

This study presents several limitations. First, the RPL sample size is relatively small, which is unlikely to capture all CNVs potentially associated with miscarriage. However, the findings still serve as a valuable addition to broader studies. Second, the limited number of WES samples resulted in a sparse distribution of SNVs, and no further functional investigations were conducted. Future studies should aim to increase the number of WES samples to concentrate on mutations within specific critical pathways. Third, although an internal healthy control group was not included, we utilized large-scale public databases (*e.g.*, DGV, gnomAD) to rigorously exclude benign population polymorphisms, strictly adhering to ACMG guidelines. Consequently, these findings identify candidate variants that contribute to the future establishment of genetic markers for the Chinese population, serving as a foundational resource rather than a definitive diagnostic panel. Lastly, the enrichment analysis of gene functions was not extensively explored, necessitating further experimental validation of the identified hub genes to elucidate their roles as genetic markers for RPL screening.

## Conclusions

In conclusion, this study utilized CNV-seq and SNP-array to investigate CNVs in RPL samples, identifying four novel gene variants through WES that may be associated with RPL. Further enrichment analysis revealed three potential hub genes. These findings contribute to the establishment of genetic markers for RPL screening in the Chinese population. This approach can enhance our understanding of miscarriage etiology, assess recurrence risk, and facilitate prenatal diagnosis.

##  Supplemental Information

10.7717/peerj.20889/supp-1 Supplemental Information 1 MIAME checklist

## Additional Information and Declarations

### Data Availability

The following information was supplied regarding data availability: The NGS results including WES and CNV-seq are available at NCBI GEO GSE310872, SRA PRJNA1364326, and Figshare: Wang, Luming (2025). All 66 SNP-array raw data and the raw data of selected abnormal CNV-seq cases. figshare. Dataset. https://doi.org/10.6084/m9.figshare.28955738.v3.
